# Dietary soluble flaxseed oils as a source of omega-3 polyunsaturated fatty acids for laying hens

**DOI:** 10.1016/j.psj.2021.101276

**Published:** 2021-05-23

**Authors:** Sang Hyeok Lee, Yoo Bhin Kim, Da-Hye Kim, Dong-Won Lee, Hong-Gu Lee, Rajesh Jha, Kyung-Woo Lee

**Affiliations:** ⁎ Department of Animal Science and Technology, Konkuk University, Gwangjin-gu, Seoul 05029, South Korea; † Haitnim Bio Inc., Icheon-si, Gyeonggi-do, 17346, South Korea; ‡ Department of Human Nutrition, Food and Animal Sciences, College of Tropical Agricultural and Human Resources, University of Hawaii at Manoa, Honolulu, HI 96822, USA

**Keywords:** soluble flaxseed oil, laying hen, laying performance, fatty acid composition, lipid oxidation

## Abstract

The present study investigated the effect of dietary soluble flaxseed oil (**SFO**), as a source of omega-3 polyunsaturated fatty acids, on the fatty acid composition of egg yolk and various indices including laying performance, egg quality, nutrient composition of eggs, egg stability upon storage, and serum characteristics in laying hens. A total of 210 52-week-old Hy-Line Brown laying hens were assigned to one of 5 experimental diets. A corn-soybean meal-based control diet was mixed without or with SFO to reach the concentrations of 0.2, 0.4, 0.6, and 0.8% in diets and fed for 4 wk. Dietary SFO did not affect laying performance and egg quality. Increasing dietary SFO linearly increased the pH of yolk at 7, 14, and 28 d following storage at room temperature (*P* < 0.05). Malondialdehyde contents in egg yolks were quadratically increased (*P* < 0.05) at 0, 7, and 21 d following storage as the inclusion levels of SFO increased in diets. A significant increase (*P* < 0.05) in total omega-3 polyunsaturated fatty acids and docosahexaenoic acid, but not α-linolenic acid and eicosapentaenoic acid, was deposited in egg yolks at 2 and 4 wk following the SFO feeding. Finally, dietary SFO did not affect serum parameters such as total cholesterol, triglyceride, high-density lipoprotein cholesterol, and nitric oxide. It is concluded that adding SFO into the diets of laying hens can be an efficient strategy to enrich the omega-3 polyunsaturated fatty acids, including docosahexaenoic acid in eggs.

## INTRODUCTION

The beneficial effects of omega-3 (**n-3**) polyunsaturated fatty acids (**PUFA**) including α-linolenic acid (**ALA**, C18:3n-3), eicosapentaenoic acid (**EPA**, C20:5n-3), and docosahexaenoic acids (**DHA**, C22:6n-3) on growth, health, and immune function for humans and animals have been well acknowledged (Cherian and Hayat, 2009; Goyal et al., 2014; Lee et al., 2019). In this sense, supplementation of various n-3 fatty acids into the diet of laying hens has been a nutritional attempt to raise the levels of n-3 PUFA in the edible eggs due to diet-mediated modification of fatty acid composition in egg yolks (Oliveira et al., 2010). The most studied dietary supplements for n-3 PUFA are fish oil, flaxseed, or microalgae (Alagawany et al., 2019). Although fish oil is rich in n-3 PUFA, especially EPA and DHA, there are negative reports that laying hens fed with fish oil-added diet produced fishy-tainted eggs and contains pollutants such as heavy metals in eggs (Lemahieu et al., 2013; Coorey et al., 2015). As a source of EPA or DHA, dietary microalgae has been recently marketed, but the relatively high production cost and fiber contents may limit its use as a feed supplement (Fraeye et al., 2012). As flaxseed is a rich source of protein (22%), oil (34%), and ALA (50% of oil) (Jia and Slominski, 2010), dietary flaxseed oil has been used to produce n-3 enriched eggs or meats in the poultry industry (Jia and Slominski, 2010; Oliveira et al., 2010; Petrović et al., 2012). However, flaxseed or flaxseed oil contains antinutritional components including cyanogenic glycosides, phytic acid, and mucilage that impair digestion and absorption of the nutrients (Fraeye et al., 2012; Ehr et al., 2017).

Recently, soluble flaxseed oil (**SFO**), as a safer food additive for human consu mption, has been marketed by the patented processes, including emulsification of refined oils with catechin-rich tea extracts and detoxification. During these processes, flaxseed oil is subjected to be emulsified and remove antinutritional factors and heavy metals. SFO is produced in either wax-like state or powdered form that is easy to mix with food and feed. With the advantages of being easy handling and the absence of antinutritional factors, ALA-rich SFO has been used as a supplement to produce n-3 PUFA-fortified milk. It is reported that emulsification of edible oils has the potential to improve the digestion and absorption of fatty acids due to the increase in solubility (Raatz et al., 2009). Indeed, Couëdelo et al. (2011) observed that ALA bioavailability was improved with the ingestion of partially emulsified flaxseed oil. It is thus expected that ALA-rich SFO could be incorporated into the eggs with high efficiency. Therefore, the aim of this study was to investigate the effect of dietary SFO on the fatty acid composition of egg yolks and various indices, including laying performance, egg quality, egg storage stability, and serum characteristics in laying hens. As PUFA in layer diets increased the susceptibility of eggs to lipid oxidation (Cherian et al., 2007), we decided to monitor yolk lipid stability upon storage as well.

## MATERIALS AND METHODS

All experimental protocols and use of laying hens in the trial were approved by the Institutional Animal Care and Use of Committee of Konkuk University (KU19051), South Korea.

### Soluble Flaxseed Oil Preparation

Dietary SFO, as an n-3-rich food/feed supplement for humans and animals, was manufactured from flaxseed via a patented solubilized process (Shin and An, 2010). The commercially available SFO was provided by Haitnim Bio, Inc. (Icheon-si, Gyeonggi-do, South Korea). Antinutrients such as cyanogenic glycosides and the odor of flaxseeds were eliminated during the roasting process (Feng et al., 2003; Yamashita et al., 2007). Machine-extracted flaxseed oil was precipitated for 10 to 20 d, and the upper layer was collected to prepare refined flaxseed oil. The 50 g of the refined flaxseed oil was homogenized with 20 g of green tea extract, 25 g of distilled water, and 5 g of emulsifier (i.e., Tween 60) at 3,000 rpm to produce 100 g of the SFO.

### Birds and Experimental Design

A total of 210 laying hens of Hy-Line Brown, aged 52 wk, were raised in a windowless chicken facility that was maintained at 22 ± 2°C for 4 wk. The basal diet was formulated (Table 1) to meet or exceed the nutrient requirements of laying hens (National Research Council, 1994). No additives, including exogenous enzymes, were included in the basal diet. Feed and water were supplied to allow ad libitum consumption during the experimental period. A lighting program of 16 h of light and 8 h of dark was used for the entire experimental period.

**Table 1:** The ingredient and nutrient composition of the basal diet.

Ingredients	g per 100 g of diet
Corn	43.0
Soybean meal, 45% crude protein	5.14
Wheat	5.59
Rice dehulled	4.0
Rice bran	2.0
Corn germ meal	5.52
Rapeseed meal	3.0
Dried distillers grains with solubles	17.0
Liquid condensed molasses solubles	1.0
Choline chloride	0.06
Limestone	10.7
Monodicalcium phosphate	0.66
NaCl	0.22
Carrier (corn)	1.25
Methionine-100%	0.06
Lysine sulfate-54%	0.25
Tryptophane-10%	0.30
Vitamin mix^1^	0.14
Mineral mix^2^	0.12
Total	100.00
Calculated nutrient composition, %	
AMEn^3^, kcal/kg	2,600
Dry matter	89.1
Crude protein	14.5
Calcium	4.13
Available phosphorus	0.28
Sodium	0.15
Chloride	0.25
Lysine	0.64
Methionine	0.32
Methionine + Cysteine	0.60
Threonine	0.52
Tryptophan	0.16

1 Vitamin mixture provided following nutrients per kg of diet: vitamin A, 15,400 IU; vitamin D_3_, 3,080 IU; vitamin E, 14 mg; vitamin K_3_, 1.4 mg; vitamin B_1_, 1.12 mg; vitamin B_2_, 2.8 mg; vitamin B_6_, 3.92 mg; vitamin B_12_, 0.014 mg; niacin, 56 mg; pantothenic acid, 5.6 mg; folic acid, 0.28 mg. biotin, 0.14 mg; choline, 260.4 mg.

2 Mineral mixture provided following nutrients per kg of diet: Mn, 70 mg; Zn, 50 mg; Fe, 50 mg; Cu, 7 mg; I, 0.75 mg; Co, 0.4 mg; Se, 0.17 mg.

3 Nitrogen-corrected apparent metabolizable energy.

The completely randomized design was applied to the feeding experiment. Laying hens were randomly allotted to one of 5 experimental diets: basal diet (control), 0.2, 0.4, 0.6, or 0.8% SFO supplementation. Two or 3 hens were housed in a cage (45 cm × 45 cm), and the adjacent 3 cages were considered a replicate (n = 7 birds/replicate, 6 replicates/treatment). Dietary SFO was added directly to the basal diet at different percentages at the expense of an equal weight of the basal diet.

### Chemical and Fatty Acid Compositions of Experimental Diets

Proximal nutrient composition of the experimental diets is presented in Table 2. Feed samples were analyzed in duplicate for moisture (Method 930.15, AOAC, 2005), ether extract (**EE**; method 920.39, AOAC, 2005), crude fiber (**CF**; method 2002.04, AOAC, 2005), neutral detergent fiber (**NDF**; Van Soest et al., 1991), acid detergent fiber (**ADF**; Van Soest et al., 1991), phosphorus (**P**; method 965.17, AOAC, 2005). The fatty acid profile of the SFO and the experimental diets (Table 3) was determined following the method described by Kim et al. (2016).

**Table 2:** The analyzed nutrient composition (as-is basis) of the experimental diets containing increasing levels of soluble flaxseed oil.^1^

Item, %	Soluble flaxseed oil concentration in diets, %				
	0	0.2	0.4	0.6	0.8
Moisture	10.81	10.93	10.83	10.68	11.14
Ether extract	3.18	3.19	3.60	3.66	3.71
Crude fiber	3.33	3.15	2.88	3.31	3.14
NDF	16.87	16.47	16.28	16.15	15.37
ADF	5.03	4.56	4.61	4.24	4.27
Phosphorus	0.60	0.55	0.54	0.57	0.47

Abbreviations: ADF, acid detergent fiber; NDF, neutral detergent fiber.

1 The average CP, ash, and calcium values for the basal diet: 14.31%, 15.52%, and 5.46%, respectively.

**Table 3:** Analyzed fatty acids composition (% of total fatty acid methyl esters) of soluble flaxseed oil and the experimental diets.

Fatty acids	Soluble flaxseed oil	Soluble flaxseed oil concentration in diets, %				
		0	0.2	0.4	0.6	0.8
Lauric acid (C12:0)	0.47	0.12	0.24	0.31	0.34	0.39
Myristic acid (C14:0)	0.04	0.20	0.24	0.27	0.29	0.32
Palmitic acid (C16:0)	4.83	15.18	15.00	14.95	15.00	14.73
Stearic acid (C18:0)	2.48	2.36	2.57	2.71	2.77	2.96
Oleic acid (C18:1n-9)	10.11	26.53	26.36	26.20	25.52	25.80
Linoleic acid (C18:2n-6)	12.78	53.47	52.26	50.33	49.48	48.17
α-linolenic acid (C18:3n-3)	69.29	2.13	3.33	5.23	6.59	7.63
Linoleic acid:α-linolenic acid	0.18	25.14	15.69	9.62	7.51	6.31

### Laying Performance Parameters and Egg Quality

Eggs were collected and weighed daily. Feed consumption per replicate was recorded and used to calculate the average daily feed intake per bird. Body weights of laying hens were recorded at the beginning and the end of the experiment. On the last 3 consecutive days at 2 and 4 wk, 6 eggs per replicate were collected for egg quality assessment. Eggshell color was estimated by shell color reflectometer (TSS QCR, Technical Services and Supplies, York, UK). Eggshell strength, yolk color score, Haugh unit, and eggshell thickness were assessed (without shell membrane) with a digital egg tester (DET−6000, Nabel, Kyoto, Japan). The separated yolks were weighed after clearing adherent albumen residues with filter paper. Eggshells were cleaned to remove the adherent albumen, dried at room temperature for 3 d, and weighed. Albumen weight was then calculated by subtracting yolk and dry eggshell weight from the initial whole egg weight. In addition, pooled eggs per replicate were analyzed for moisture (Method 930.15, AOAC, 2005), CP **(**Method 990.03, AOAC, 2005), EE (Method 920.39, AOAC, 2005), and ash (Method 942.05, AOAC, 2005).

### Measurement of pH and Lipid Oxidation

For the measurement of pH and lipid oxidation upon storage, fresh eggs were sampled during the last 3 consecutive days in the wk 4 and then stored at room temperature (i.e., 25°C) for 28 d. At 0, 7, 14, 21, and 28 d upon storage, the pH of yolk and albumen (3 eggs/replicate) was measured using a pH meter (HI98163, Hanna Instrument Inc., Woonsocket, RI). Immediately after pH measurement, yolks were separated and measured for malondialdehyde (**MDA**) contents as the indicator of yolk lipid oxidation using the OxiSelect TBARS Assay kit (Cell Biolabs, Inc., San Diego, CA).

### Analysis of Fatty Acid Composition

At 2 and 4 wk, 2 eggs per replicate were used for fatty acid analysis. Eggs were weighed and yolks were separated using an egg yolk separator. Yolk weights were recorded and subjected to measurement of fatty acid composition. In brief, approximately 1 g of pooled egg yolks was extracted with chloroform:methanol (2:1, vol/vol) mixture according to the method of Folch et al. (1957). Tridecanoic acid (C13:0) was used as an internal standard. The fatty acids in extracted lipids were then transesterified by boron-trifluoride to fatty acid methyl esters (**FAMEs**). The FAMEs separated were analyzed by gas chromatography (HP 6890 series GC system, Agilent Technologies, Palo Alto, CA) equipped with an SP-2560 capillary column (dimension: 100 meter × 0.25 mm × 0.2 µm) and a flame ionization detector. The initial column temperature was set at 70°C for 5 min, increased to 175°C, and held for 10 min. The column temperature was elevated to 225°C and held at the final temperature for 5 min. Helium was used as the carrier gas at a flow rate of 1.2 mL/min. The detector was set at 260°C. FAMEs were identified by comparison with retention times of the standard. FAME Mix STANDARD (Sigma-Aldrich/47885-U, St. Louis, MO) was the standard for fatty acid content and fatty acid ratio. Identified fatty acids were expressed as a percentage of total fatty acids. Additionally, ALA (C18:3n-3), EPA (C20:5n-3), and DHA (C22:6n-3) of egg yolk were quantified according to their percentage area, obtained by integration of the peak. For the determination of fatty acid profiles in SFO and the experimental diets, approximately 1 g of samples was used as described above.

### Serum Biochemical Parameters and Nitric Oxide Measurement

At the end of the experiment, blood was taken from the wing vein of one hen per replicate. Serum was obtained following centrifugation at 200 × g for 15 min and stored at −20°C until further analysis. Serum samples were analyzed for glutamic pyruvic transaminase (**GPT**), glutamic oxaloacetic transaminase (**GOT**), high-density lipoprotein cholesterol (**HDL**), total cholesterol (**TCH**), and triglyceride (**TG**) using an automatic blood chemical analyzer (Film DRI CHEM 7000i, Fuji film, Tokyo, Japan). The nitric oxide (**NO**) concentrations in serum samples were determined as described by Lee et al., (2011). NO concentration was calculated from a standard curve with sodium titrate as described by Lee et al., (2011).

### Statistical Analysis

Data were analyzed using the general linear model procedures of SAS 9.4 (SAS Inst. Inc., Cary, NC) in a completely randomized design. Three adjacent cages were considered as an experimental unit. Orthogonal polynomial contrasts were used to determine the linear and quadratic effects of graded levels of dietary SFO. Tukey post hoc tests were performed when significant differences were found. Significant differences were considered at *P* < 0.05 and tendency was declared at *P* < 0.10.

## RESULTS

### Analyzed Fatty Acids Compositions of Soluble Flaxseed Oil and Experimental Diets

As expected, the SFO had the highest ALA content (69.3% of total FAMEs, Table 3). The ALA contents in the experimental diets were gradually increased with increasing levels of SFO into the basal diet (Table 3). The ALA content increased from 2.13% to 7.63%, but linoleic acid content decreased from 53.47% to 48.17%, leading to the ratio of linoleic acid and ALA decreasing from 25.14 to 6.31 (Table 3).

### Laying Performance and Body Weight

Production performance in laying hens fed diets with varying SFO levels is presented in Table 4. There were no significant (*P* > 0.05) linear and quadratic effects of dietary SFO levels on feed intake, egg production, egg weight, egg mass, and feed to egg ratio. The average body weight of the hens was 1.89 ± 0.086 kg at the end of the experiment, with no significant (*P* > 0.05) difference among treatments.

**Table 4:** Effect of dietary soluble flaxseed oil on laying performance and body weight in laying hens.

Item	Soluble flaxseed oil concentration in diets, %					SEM	*P-*value		
	0	0.2	0.4	0.6	0.8		ANOVA	Linear	Quadratic
Average daily feed intake, g	124.46	126.03	124.60	124.45	122.92	2.81	0.959	0.612	0.701
Egg production, %	76.61	78.81	73.21	76.87	80.04	3.55	0.856	0.671	0.494
Egg weight, g	62.69	61.89	62.96	61.32	63.01	0.76	0.565	0.975	0.513
Egg mass, (g/d/bird)	48.07	48.75	46.09	47.18	50.47	2.40	0.848	0.681	0.419
Feed to egg ratio^1^, (g:g)	2.62	2.61	2.70	2.66	2.44	0.10	0.549	0.363	0.235
Body weight, kg/bird									
D 0	1.82	1.88	1.89	1.87	1.81	0.05	0.657	0.852	0.134
D 28	1.87	1.90	1.91	1.93	1.85	0.04	0.555	0.801	0.143

Abbreviation: ANOVA, analysis of variance.

1 Feed to egg ratio, average daily feed intake/egg mass.

### Egg Components and Egg Quality

Increasing SFO levels in the diets did not influence (*P* > 0.05) none of the variables in egg components and egg quality, albeit that relative eggshell weight tended (*P* = 0.078 at 2 wk, *P* = 0.119 at 4 wk) to increase with increasing dietary SFO (Table 5).

**Table 5:** Effect of dietary soluble flaxseed oil on egg components and eggshell quality in laying hens.

Item	Soluble flaxseed oil concentration in diets, %					SEM	*P-*value		
	0	0.2	0.4	0.6	0.8		ANOVA	Linear	Quadratic
After 2 wk of feeding									
Relative yolk weight, %	25.95	25.73	26.64	26.10	25.70	0.40	0.476	0.917	0.235
Relative eggshell weight, %	9.90	9.73	9.79	10.06	10.12	0.13	0.201	0.078	0.188
Relative albumen weight, %	64.13	64.54	63.55	63.84	64.24	0.42	0.524	0.718	0.431
Yolk color	7.01	6.85	6.92	6.95	6.91	0.16	0.970	0.846	0.720
Haugh unit	77.49	78.03	79.01	79.89	78.69	1.91	0.917	0.487	0.619
Eggshell strength, kg/cm^2^	4.37	4.26	4.21	4.33	4.47	0.14	0.735	0.563	0.217
Eggshell thickness, mm	0.41	0.41	0.41	0.42	0.40	0.01	0.611	0.864	0.499
Eggshell color, unit	31.94	31.58	33.25	32.28	32.11	0.88	0.733	0.715	0.500
After 4 wk of feeding									
Relative yolk weight, %	25.71	25.76	25.61	26.43	25.25	0.38	0.303	0.831	0.301
Relative eggshell weight, %	9.96	9.91	9.92	10.04	10.08	0.07	0.403	0.119	0.316
Relative albumen weight, %	63.99	64.57	64.62	63.84	64.63	0.42	0.518	0.686	0.796
Yolk color	7.29	7.43	7.36	7.15	7.28	0.15	0.774	0.553	0.776
Haugh unit	74.47	78.63	75.66	76.27	76.69	2.02	0.687	0.750	0.612
Eggshell strength, kg/cm^2^	4.48	4.47	4.38	4.43	4.59	0.12	0.800	0.668	0.291
Eggshell thickness, mm	0.40	0.39	0.39	0.40	0.40	0.00	0.355	0.497	0.126
Eggshell color, unit	31.14	32.94	31.42	31.58	30.94	0.76	0.384	0.471	0.269

Abbreviation: ANOVA, analysis of variance.

### Proximate Composition of Egg

The effect of dietary SFO levels on proximate compositions of eggs (egg yolk plus albumen) is presented in Table 6. At 2 wk, increasing dietary SFO levels linearly decreased (*P* = 0.001) crude protein but linearly increased (*P* = 0.021) ether extract of eggs. On the other hand, dietary treatments did not affect moisture and ash contents. No linear or quadratic effect of dietary SFO levels on proximate compositions at 4 wk was noted. However, concentrations of ether extract in eggs were highest in hens fed a 0.6% SFO-added diet but lowest in hens fed a 0.4% SFO-added diet.

**Table 6:** Nutrient composition (as-is basis) of whole eggs from laying hens fed diets containing increasing levels of soluble flaxseed oil.

Item	Soluble flaxseed oil concentration in diets, %					SEM	*P-*value		
	0	0.2	0.4	0.6	0.8		ANOVA	Linear	Quadratic
After 2 wk of feeding									
Moisture, %	78.88	79.02	79.52	78.53	78.21	0.50	0.431	0.255	0.211
Crude protein, %	11.72^a^	10.69^bc^	10.81^bc^	11.00^b^	10.27^c^	0.22	0.002	0.001	0.442
Ether extract, %	6.14	6.07	6.22	6.83	6.87	0.28	0.143	0.021	0.521
Ash, %	0.87	0.85	0.91	0.88	0.85	0.02	0.337	0.981	0.252
After 4 wk of feeding									
Moisture, %	76.73	76.86	77.53	75.91	76.48	0.38	0.071	0.234	0.327
Crude protein, %	11.80	12.10	11.70	11.65	11.84	0.12	0.129	0.366	0.794
Ether extract, %	8.07^bc^	8.48^ab^	7.71^c^	8.73^a^	8.12^bc^	0.15	0.001	0.461	0.658
Ash, %	0.89	0.98	0.93	1.00	0.95	0.03	0.070	0.112	0.116

^a,b,c^Mean value without a common superscript within the same row differ (*P* < 0.05).

Abbreviation: ANOVA, analysis of variance.

### Change in Egg pH and Yolk MDA Upon Storage

Graded SFO levels did not affect the pH of the albumen upon storage (Table 7). On the other hand, the pH of the yolk at 7, 14, and 28 d following storage was linearly increased with increasing dietary SFO levels. Of interest, the concentration of MDA as an indicator of lipid peroxidation displayed quadratic decrease (*P* < 0.05) at 0, 7, and 21 d following storage as dietary SFO levels increased in the diets. The ANOVA analysis revealed that this quadratic pattern in MDA contents was mainly due to the highest MDA contents in 0.8% SFO-added diet-fed groups.

**Table 7:** Effects of dietary soluble flaxseed oil on albumen and yolk pH and malondialdehyde in yolk during storage at room temperature.

Item	Soluble flaxseed oil concentration in diets, %					SEM	*P-*value		
	0	0.2	0.4	0.6	0.8		ANOVA	Linear	Quadratic
Albumen pH									
0 d	8.48	8.40	8.45	8.43	8.46	0.04	0.569	0.851	0.261
7 d	9.39	9.46	9.44	9.41	9.44	0.04	0.747	0.717	0.511
14 d	9.45	9.42	9.39	9.45	9.43	0.03	0.668	0.932	0.349
21 d	9.59	9.54	9.45	9.58	9.44	0.05	0.136	0.118	0.826
28 d	9.51	9.57	9.41	9.54	9.43	0.05	0.192	0.263	0.848
Yolk pH									
0 d	5.87	5.88	5.83	5.78	5.92	0.05	0.259	0.942	0.132
7 d	6.02^c^	6.10^bc^	6.25^ab^	6.29^a^	6.36^a^	0.06	0.001	<0.001	0.492
14 d	5.91	5.89	6.16	6.02	6.09	0.07	0.053	0.036	0.368
21 d	6.21	6.12	6.15	6.12	6.20	0.05	0.566	0.967	0.143
28 d	6.25^b^	6.25^b^	6.20^b^	6.34^ab^	6.54^a^	0.08	0.040	0.013	0.056
MDA, µM									
0 d	48.53^ab^	42.99^b^	43.40^b^	43.66^b^	54.21^a^	1.92	0.029	0.132	0.004
7 d	52.03	49.52	51.72	49.55	56.74	1.65	0.069	0.133	0.049
14 d	56.71	50.32	50.71	50.23	63.75	7.85	0.697	0.619	0.246
21 d	58.15^b^	51.79^b^	52.27^b^	54.89^b^	66.40^a^	1.51	0.013	0.024	0.003
28 d	69.18	62.05	62.92	77.60	74.16	4.14	0.223	0.125	0.248

^a,b,c^Mean value without a common superscript within the same row differ (*P* < 0.05).

Abbreviations: ANOVA, analysis of variance; MDA, malondialdehyde.

### Fatty Acid Composition of Egg Yolks

The fatty acids composition of egg yolks at 2 wk (expressed as a percentage of total FAMEs) are shown in Table 8. Increasing dietary SFO linearly decreased (*P* < 0.05) the percentages of palmitic acid, α-linolenic acid, and nervonic acid, but linearly increased (*P* < 0.05) the percentages of heptadecanoic acid, eicosatrienoic acid, and DHA in egg yolks. The concentrations of total PUFAs and total n-3 fatty acids in egg yolks were significantly affected (linear and quadratic effect, *P* < 0.05) by dietary SFO levels. The ratio of n-3 to n-6 fatty acids in egg yolks increased (linear and quadratic effect, *P* < 0.05) with increasing SFO levels in the diets. The fatty acid composition of egg yolks laid at 4 wk (expressed as the percentage of total FAMEs) exhibited a similar pattern as seen at 2 wk, but the affected fatty acids to dietary SFO levels were lesser at 4 wk. Increasing dietary SFO levels decreased (*P* < 0.05) the percentage of α-linolenic acid but increased (*P* < 0.05) that of eicosatrienoic acid and DHA in egg yolks. Total n-3 fatty acids and the ratio of n-3 to n-6 fatty acids were significantly increased with increasing dietary SFO levels (Table 9).

**Table 8:** Fatty acids composition (% of total fatty acid methyl esters) of egg yolk from laying hens fed diets containing increasing levels of soluble flaxseed oil for 2 wk.^1^

Fatty acids	Soluble flaxseed oil concentration in diets, %					SEM	*P-*value		
	0	0.2	0.4	0.6	0.8		ANOVA	Linear	Quadratic
Palmitic acid (C16:0)	26.75^ab^	26.49abc	26.88^a^	25.44^c^	25.68^bc^	0.37	0.034	0.012	0.558
Heptadecanoic acid (C17:0)	0.14^c^	0.16^bc^	0.16^bc^	0.19^a^	0.17^ab^	0.01	0.004	0.001	0.111
Oleic acid (C18:1n-9)	41.81	39.75	38.86	39.58	41.03	1.09	0.341	0.617	0.045
Linoleic acid (C18:2n-6)	14.76^b^	16.84^a^	15.65^ab^	17.01^a^	15.85^ab^	0.46	0.012	0.118	0.031
γ-linolenic acid (C18:3n-6)	0.11	0.13	0.11	0.12	0.11	0.01	0.120	0.694	0.186
α-linolenic acid (C18:3n-3)	0.25^b^	0.38^a^	0.39^a^	0.18^b^	0.19^b^	0.02	<0.001	<0.001	<0.001
Dihomo-γ-linolenic acid (C20:3n-6)	0.18	0.17	0.19	0.17	0.16	0.01	0.747	0.513	0.400
Eicosatrienoic acid (C20:3n-3)	0.01^b^	0.02^b^	0.02^a^	0.03^a^	0.03^a^	0.00	<0.001	<0.001	0.264
Arachidonic acid (C20:4n-6)	0.01^abc^	0.01^a^	0.01^ab^	0.01^bc^	0.01^c^	0.00	0.009	0.007	0.019
Eicosapentaenoic acid (C20:5n-3)	0.01	0.01	0.02	0.01	0.01	0.00	0.659	0.630	0.166
Docosahexaenoic acid (C22:6n-3)	0.66^e^	1.02^d^	1.28^c^	1.64^a^	1.45^b^	0.04	<0.001	<0.001	<0.001
Nervonic acid (C24:1n-9)	0.25^a^	0.13^b^	0.18^b^	0.19^ab^	0.05^c^	0.02	<0.001	0.000	0.410
Others^2^	15.06	14.88	15.97	15.43	15.14	0.62	0.753	0.717	0.430
Total SFA^3^	35.87	35.63	36.82	34.87	34.81	0.68	0.239	0.192	0.279
Total MUFA^4^	48.00	45.63	45.08	45.77	47.11	0.93	0.188	0.582	0.020
Total PUFA^5^	16.13^c^	18.74^ab^	17.91^ab^	19.36^a^	17.69^ab^	0.49	0.001	0.029	0.003
Total n-9^6^	44.72	42.61	42.25	43.06	44.24	0.91	0.272	0.860	0.031
Total n-6^7^	15.05^b^	17.15^a^	15.96^ab^	17.31^a^	16.13^ab^	0.47	0.013	0.130	0.030
Total n-3^8^	0.93^d^	1.43^c^	1.74^ab^	1.86^a^	1.69^b^	0.05	<0.001	<0.001	<0.001
n-3:n-6 ratio^9^	0.06^c^	0.08^b^	0.11^a^	0.11^a^	0.11^a^	0.00	<0.001	<0.001	<0.001

Abbreviation: ANOVA, analysis of variance.

a,b,c Mean value without a common superscript within the same row differ (*P* < 0.05).

1 Data were reported as % of total fatty acid methyl esters.

2 Others were calculated as C15:0 + C16:1 + C17:1 + C18:0 + C20:1n-9 + C20:2 + C22:1n-9.

3 SFA, saturated fatty acids; SFA level were calculated as C15:0 + C16:0 + C17:0 + C18:0.

4 MUFA, monounsaturated fatty acids; MUFA levels were calculated as C16:1 + C17:1 + C18:1n-9 + C20:1n-9 + C22:1n-9 + C24:1n-9.

5 PUFA, polyunsaturated fatty acids; PUFA levels were calculated as C18:2n-6 + C18:3n-6 + C18:3n-3 + C20:2 + C20:3n-6 + C20:3n-3 + C20:4n-6 + C20:5n-3 + C22:6n-3.

6 Total n-9 was calculated as C18:1n-9 + C20:1n-9 + C22:1n-9 + C24:1n-9.

7 Total n-6 was calculated as C18:2n-6 + C18:3n-6 + C20:3n-6 + C20:4n-6.

8 Total n-3 was calculated as C18:3n-3 + C20:3n-3 + C20:5n-3 + C22:6n-3.

9 Ratio of n-3:n-6 was calculated as Total n-3:Total n-6.

**Table 9:** Fatty acids composition (% of total fatty acid methyl esters) of egg yolk from laying hens fed diets containing increasing levels of soluble flaxseed oil for 4 wk.^1^

Fatty acids	Soluble flaxseed oil concentration in diets, %					SEM	*P-*value		
	0	0.2	0.4	0.6	0.8		ANOVA	Linear	Quadratic
Palmitic acid (C16:0)	26.98	26.65	27.07	26.70	26.76	0.40	0.928	0.762	0.994
Heptadecanoic acid (C17:0)	0.13	0.13	0.14	0.13	0.13	0.01	0.786	0.674	0.355
Oleic acid (C18:1n-9)	42.96	42.45	42.15	43.05	41.67	0.68	0.590	0.364	0.837
Linoleic acid (C18:2n-6)	13.69	13.88	13.48	13.11	13.51	0.57	0.904	0.533	0.839
γ-linolenic acid (C18:3n-6)	0.10	0.10	0.10	0.09	0.10	0.00	0.421	0.477	0.600
α-linolenic acid (C18:3n-3)	0.23	0.21	0.21	0.21	0.20	0.01	0.298	0.039	0.709
Dihomo-γ-linolenic acid (C20:3n-6)	0.17	0.18	0.17	0.16	0.17	0.01	0.305	0.260	0.513
Eicosatrienoic acid (C20:3n-3)	0.01^d^	0.02^c^	0.02^bc^	0.02^b^	0.03^a^	0.00	<0.001	<0.001	0.234
Arachidonic acid (C20:4n-6)	0.01	0.01	0.01	0.01	0.01	0.00	0.297	0.052	0.350
Eicosapentaenoic acid (C20:5n-3)	0.01	0.01	0.01	0.01	0.01	0.00	0.907	0.848	0.936
Docosahexaenoic acid (C22:6n-3)	0.53^e^	0.77^d^	1.02^c^	1.14^b^	1.32^a^	0.03	<0.001	<0.001	0.038
Nervonic acid (C24:1n-9)	0.51	0.55	0.57	0.52	0.50	0.02	0.052	0.425	0.008
Others^2^	14.67^b^	14.99^ab^	15.00^ab^	14.82^b^	15.51^a^	0.18	0.034	0.014	0.416
Total SFA^3^	35.93	35.63	36.09	35.46	35.71	0.37	0.764	0.603	0.999
Total MUFA^4^	49.17	48.99	48.70	49.62	48.72	0.64	0.837	0.897	0.927
Total PUFA^5^	14.90	15.05	14.87	14.69	15.35	0.59	0.961	0.789	0.664
Total n-9^6^	45.74	45.47	45.23	46.04	45.00	0.62	0.781	0.651	0.831
Total n-6^7^	13.97	14.17	13.77	13.37	13.79	0.57	0.893	0.520	0.838
Total n-3^8^	0.78^e^	1.01^d^	1.26^c^	1.38^b^	1.57^a^	0.03	<0.001	<0.001	0.100
n-3:n-6 ratio^9^	0.06^d^	0.07^c^	0.09^b^	0.11^ab^	0.12^a^	0.00	<0.001	<0.001	0.149

^a,b,c^Mean value without a common superscript within the same row differ (*P* < 0.05).

Abbreviation: ANOVA, analysis of variance.

1 Data were reported as % of total fatty acid methyl esters.

2 Others were calculated as C15:0 + C16:1 + C17:1 + C18:0 + C20:1n-9 + C20:2 + C22:1n-9.

3 SFA = saturated fatty acids; SFA level were calculated as C15:0 + C16:0 + C17:0 + C18:0.

4 MUFA, monounsaturated fatty acids; MUFA levels were calculated as C16:1 + C17:1 + C18:1n-9 + C20:1n-9 + C22:1n-9 + C24:1n-9.

5 PUFA, polyunsaturated fatty acids; PUFA levels were calculated as C18:2n-6 + C18:3n-6 + C18:3n-3 + C20:2 + C20:3n-6 + C20:3n-3 + C20:4n-6 + C20:5n-3 + C22:6n-3.

6 Total n-9 was calculated as C18:1n-9 + C20:1n-9 + C22:1n-9 + C24:1n-9.

7 Total n-6 was calculated as C18:2n-6 + C18:3n-6 + C20:3n-6 + C20:4n-6.

8 Total n-3 was calculated as C18:3n-3 + C20:3n-3 + C20:5n-3 + C22:6n-3.

9 Ratio of n-3:n-6 was calculated as Total n-3:Total n-6.

### Omega-3 Polyunsaturated Fatty Acid Contents in Egg Yolks

As dietary SFO affected the percentage of egg fatty acid composition, we further quantified n-3 PUFA contents in egg yolks (Table 10). At 2 wk, ALA contents increased from 24.7 mg/yolk in no SFO-added control diet-fed group to 43.7 mg/yolk in 0.4% SFO-added diet-fed group, and then declined to approximately 20 mg/yolk in laying hens fed higher SFO-added diets. Dietary SFO did not affect EPA contents that were ranged from 1.06 to 1.46 mg/yolk. Total n-3 PUFA and DHA contents increased (linear and quadratic effect, *P* < 0.05) with increasing dietary SFO levels and plateaued being 131.5 mg DHA/yolk and 152.9 mg n-3 PUFA in 0.6% SFO-fed laying hens. At 4 wk, ALA and EPA contents ranged from 19.6 to 23.1 mg/yolk and 1.08 to 1.49 mg/yolk. Total n-3 PUFA and DHA contents linearly increased with increasing dietary SFO levels, but their quantities in yolks were plateaued within the dietary inclusion levels being 109.1 mg DHA and 131.8 mg n-3 PUFA per yolk in laying hens fed 0.8% SPO-added diet.

**Table 10:** Omega-3 polyunsaturated fatty acids contents in yolk (mg/yolk) from laying hen fed diets containing increasing levels of soluble flaxseed oil.

Fatty acids^1^	Soluble flaxseed oil concentration in diets, %					SEM	*P-*value		
	0	0.2	0.4	0.6	0.8		ANOVA	Linear	Quadratic
After 2 wk of feeding									
α-linolenic acid (C18:3n-3)	24.74^c^	35.66^b^	43.72^a^	20.00^c^	19.50^c^	1.82	<0.001	<0.001	<0.001
Eicosapentaenoic acid (C20:5n-3)	1.24	1.46	1.27	1.34	1.06	0.11	0.212	0.195	0.109
Docosahexaenoic acid (C22:6n-3)	48.83^e^	70.24^d^	93.62^c^	131.54^a^	112.13^b^	5.40	<0.001	<0.001	0.003
Total n-3	74.81^c^	107.36^b^	140.85^a^	152.88^a^	132.69^a^	6.46	<0.001	<0.001	<0.001
After 4 wk of feeding									
α-linolenic acid (C18:3n-3)	22.93	20.99	19.59	23.12	21.37	1.30	0.315	0.814	0.287
Eicosapentaenoic acid (C20:5n-3)	1.49	1.22	1.08	1.19	1.42	0.12	0.142	0.652	0.012
Docosahexaenoic acid (C22:6n-3)	39.54^c^	58.91^b^	72.05^b^	97.21^a^	109.05^a^	5.52	<0.001	<0.001	0.884
Total n-3	63.96^c^	81.12^bc^	92.73^b^	121.51^a^	131.84^a^	6.02	<0.001	<0.001	0.877

^a,b,c^Mean value without a common superscript within the same row differ (*P* < 0.05).

Abbreviation: ANOVA, Analysis of variance.

1 Total n-3 was calculated as C18:3n-3 + C20:5n-3 + C22:6n-3.

### Serum Immunological and Biochemical Parameters

Dietary SFO did not affect any of the serum immunological and biochemical parameters studied (GOT, GPT, TCH, TG, HDL, and NO, Table 11).

**Table 11:** Effect of dietary soluble flaxseed oil on serum biochemical parameters in laying hens.

Item^1^	Soluble flaxseed oil concentration in diets, %					SEM	*P-*value		
	0	0.2	0.4	0.6	0.8		ANOVA	Linear	Quadratic
GOT, IU/L	119.7	136.2	124.0	114.3	117.5	7.13	0.256	0.257	0.373
GPT, IU/L	2.00	2.50	2.67	1.83	2.00	0.25	0.121	0.414	0.092
TCH, mg/dl	116.5	123.7	129.0	112.5	115.8	9.24	0.699	0.673	0.386
TG, mg/dl	2023	2029	2069	2074	2153	116.49	0.953	0.466	0.813
HDL, mg/dl	5.17	6.33	3.00	4.83	5.17	1.61	0.695	0.771	0.567
NO, µM	132.7	140.8	153.6	150.1	174.5	30.99	0.932	0.421	0.901

Abbreviation: ANOVA, analysis of variance.

1 GOT, glutamic oxaloacetic transaminase; GPT, glutamic pyruvic transaminase; TCH, total cholesterol; TG, triglyceride; HDL, high density lipoprotein cholesterol; NO, nitric oxide.

## DISCUSSION

Fat is used in poultry diets to decrease dustiness and enhance energy density. It is well documented that dietary fats affect egg yolk lipid composition depending upon their unsaturated or saturated fatty acid contents (Celebi and Macit, 2009). The n-3 PUFAs are considered essential fatty acids for poultry and therefore have to be provided by diet (Dunbar et al., 2014; Beheshti Moghadam and Cherian, 2017). The beneficial effect of dietary n-3 PUFA for human consumption has been well documented, and the health benefit of intaking n-3 PUFA-enriched foods includes the prevention of cardiovascular diseases and inflammatory diseases (Yi et al., 2015; Lee et al., 2019). The principal biological role of ALA is to serve as a substrate for the synthesis of n-3 PUFA such as EPA and DHA (Fraeye et al., 2012; Liang et al., 2017). Laying hens have the ability to elongate and desaturate ALA, the predominant n-3 PUFA in flaxseed, to the functional EPA and DHA (Ehr et al., 2017). Jia et al. (2008) and Samman et al. (2009) reported that adding dietary flaxseed can increase n-3 PUFA, including ALA, EPA, and DHA contents in eggs. However, the most critical problem in ALA-rich flaxseed oil is the presence of antinutrients that may have an adverse influence on health (Beheshti Moghadam and Cherian, 2017). Antinutritional factors, including cyanogenic glycosides and tannin in flaxseed, lower laying performance, and egg quality and are responsible for abnormal respiration and nervousness of laying hen (Imran et al., 2015). Dietary SFO is produced with refined flaxseed oil via a patented process including roasting, separation, and mixing with plant extracts, and SFO is being exposed more to pancreatic lipase leading to efficient digestion and absorption of n-3 PUFAs (Raatz et al., 2009). Thus, it was anticipated that dietary SFO being soluble (i.e., emulsified) would increase the incorporation of n-3 PUFA in eggs and affect overall laying performance and egg qualities.

In this study, dietary SFO did not affect laying performance (Table 4) and egg qualities (Table 5) but modified the nutrient composition of eggs. In line with this study, no effects of dietary n-3 PUFA on laying performance and egg qualities were reported (García-Rebollar et al., 2008; Neijat et al., 2016; Beheshti Moghadam et al., 2020). It should be kept in mind that we added lower amounts of SFO ranging from 0.2 to 0.8% in diets compared with the previous studies being the inclusion levels ranging from 0.5 to 5.0% in diets (Petrović et al., 2012; Ehr et al., 2017). In this study, SFO-fed laying hens exhibited a linear decrease in crude protein but the linear increase in ether extracts in eggs following 2 wk of feeding (Table 6). This finding suggests that diet-origin fat might be efficiently deposited into eggs, thus substituting crude protein contents in egg composition as dietary SFO levels increased. Similar results have been reported in laying hens previously (Ceylan et al., 2011). In contrast to our finding, Yi et al. (2014) failed to see the modification of the crude fat and crude protein composition of the eggs by dietary flaxseed oil. The failure of proximate composition of eggs might be due to the equal amount of soybean oil and flaxseed oil used in the study by Yi et al. (2014).

Yolk and albumen are enclosed by the eggshell, which allows an exchange of carbon dioxide, oxygen, and moisture via pores upon storage (Vlčková et al., 2019). Due to the loss of carbon dioxide, extended storage of eggs can increase the pH of albumen and yolk (Grčević et al., 2019; Pires et al., 2020). In this study, increasing dietary SFO levels did not affect the pH of albumen but increased the pH of yolk at 7, 14, and 28 d following storage. In contrast to our finding, Cedro et al. (2009) and Adabi et al. (2016) reported that n-3 PUFA supplementation did not affect the pH of yolk and albumen upon storage. At this stage, a clear explanation of the SFO-mediated increase in the pH of yolk, but not albumen, is not available that needs to be answered.

Lipid oxidation is a process that affects egg yolk lipid stability during storage (Omri et al., 2019) and affects egg quality, particularly its taste, flavor, odor, color, and nutritional value (Faitarone et al., 2016; Omri et al., 2019). The inclusion of PUFA in the diets of laying hens is known to increase the susceptibility of eggs to lipid oxidation (Faitarone et al., 2016). Furthermore, PUFA-rich eggs are more susceptible to oxidation as PUFAs have several double bonds (Wang et al., 2017). In this study, we monitored the MDA contents as the marker for lipid peroxidation (Rostami et al., 2016; Table 7). Of interest, increasing dietary SFO exhibited the quadratic decrease in the MDA levels of egg yolks at 0, 7, and 21 d following storage. This quadratic pattern was mainly due to the highest MDA levels in 0.8% SFO-fed laying hens. Thus, preventive nutritional measures, including dietary antioxidants, are required to inhibit lipid peroxidation when dietary SFO is added into the diets of laying hens at the levels of 0.8% or higher.

DHA has a beneficial effect on diseases such as hypertension, arthritis, atherosclerosis, depression, adult-onset diabetes mellitus, myocardial infarction, thrombosis, and some cancers (Horrocks and Yeo, 1999). It also plays a vital role in the structure and function of the brain and eye, which needs adequate supply during fetal or infant stages for optimal development (Calder, 2016). It is clear from this study that increasing dietary SFO levels effectively increased n-3 PUFA and DHA, but not ALA and EPA contents, in egg yolks (Table 8 and Table 9). Our findings suggest that diet-origin ALA is efficiently converted to long-chain n-3 PUFA (i.e., DHA) via an elongation and desaturation pathway in hepatocytes (Cherian and Sim, 1991). In addition, higher efficient conversion of EPA to DHA has been well established in laying hens, explaining the negligible amounts of EPA vs. DHA (Beynen, 2004). It has been reported that it takes on average 2 wk for laying hens to adjust to an n-3 fatty acid-enriched diet and reach a transfer plateau of diet-origin n-3 PUFA into developing ovarian follicles (Ehr et al., 2017). We also observed that increasing levels of dietary SFO consistently increased n-3 PUFA and DHA in eggs laid at 2 and 4 wk of feeding.

Furthermore, we quantified n-3 PUFA contents in yolks, including ALA, EPA, and DHA (Table 10). DHA contents ranged from 48.8 to 131.5 mg/yolk at 2 wk and from 39.5 to 109.1 mg/yolk at 4 wk. The detected DHA levels deposited into egg yolks by dietary SFO are considered higher compared to those reported elsewhere with ground flaxseed or flaxseed oils. Neijat et al. (2016) observed that the DHA content was ranged from 50.75 to 69.45 mg/yolk when laying hens were fed diets containing graded levels of flaxseed oils at the levels of 0.0 to 0.6%. Bean and Leeson (2003) reported that diets containing flaxseed at 10% increased the contents of DHA in egg yolks from 53.3 to 83.7 mg/50-g egg, while ALA increased from 38.5 to 306.3 mg/50-g egg. Aymond and Van Elswyk (1995) reported that laying hens fed diets containing EPA/DHA-rich menhaden oil at 1.5% and ALA-rich ground flaxseed at 15% deposited DHA at the concentrations of 83 mg and 90 mg/egg, respectively. Lawlor et al. (2010) reported that DHA contents were deposited to be 96 and 129 mg/total yolk in laying hens fed 2 and 4% microencapsulated fish oil-added diets. Due to inherent low conversion efficiency from ALA to DHA in humans, the WHO recommends an intake of more 250 mg DHA plus EPA daily for adults (Thompson et al., 2019). Thus, consuming an egg daily can satisfy approximately 30 to 50% of DHA requirements for adults. Further studies are warranted to clarify how dietary SFO would efficiently increase digestion and absorption of the n-3 PUFAs, and their efficient conversion from diet-origin ALA to long-chain n-3 PUFA such as EPA and DHA.

In conclusion, dietary ALA-rich SFO did not affect laying performance and egg quality but affected nutrient composition (protein and ether extracts) of eggs. In addition, the MDA contents, as the indicator of lipid peroxidation, in eggs following storage at room temperature were increased especially in groups fed 0.8% SFO-fed diets compared with the control-diet-fed laying hens. Finally, increasing levels of dietary SFO did not affect ALA content but increased n-3 PUFA and DHA in egg yolks. Thus, dietary SFO can be considered as a functional feed additive to enrich DHA in fresh eggs.

## DISCLOSURES

No potential conflict of interest was reported by the authors.

## References

[bib0001] Adabi S.G., Fani A., Ceylan N., Hajibabaei A., Casey N.H. (2016). Enrichment of quail (Coturnix cot. Japonica) eggs by omega-3 fatty acids and its nutritional effect on young healthy women. Eur. Poult. Sci..

[bib0002] Alagawany M., Elnesr S.S., Farag M.R., Abd El-Hack M.E., Khafaga A.F., Taha A.E., Tiwari R., Iqbal Yatoo M., Bhatt P., Khurana S.K., Dhama K. (2019). Omega-3 and omega-6 fatty acids in poultry nutrition: effect on production performance and health. Animals.

[bib0003] AOAC (2005). Official Methods of Analysis.

[bib0004] Aymond W.M., Van Elswyk M.E. (1995). Yolk thiobarbituric acid reactive substances and n-3 fatty acids in response to whole and ground flaxseed. Poult. Sci..

[bib0005] Bean L.D., Leeson S. (2003). Long-term effects of feeding flaxseed on performance and egg fatty acid composition of brown and white hens. Poult. Sci..

[bib0006] Beheshti Moghadam M.H., Cherian G. (2017). Use of flaxseed in poultry feeds to meet the human need for n-3 fatty acids. Worlds. Poult. Sci. J..

[bib0007] Beheshti Moghadam M.H., Shehab A., Cherian G. (2020). Production performance, quality and lipid composition of eggs from laying hens fed heated flaxseed with carbohydrase enzymes. J. Appl. Poult. Res..

[bib0008] Beynen A.C. (2004). Fatty acid composition of eggs produced by hens fed diets containing groundnut, soya bean or linseed. NJAS - Wageningen J. Life Sci..

[bib0009] Calder P.C. (2016). Docosahexaenoic acid. Ann. Nutr. Metab..

[bib0010] Cedro T.M.M., Calixto L.F.L., Gaspar A., Curvello F.A., Hora A.S. (2009). Internal quality of conventional and omega-3-enriched commercial eggs stored under different temperatures. Brazilian J. Poult. Sci..

[bib0011] Celebi S., Macit M. (2009). Effects of feeding tallow and plant fat to laying hens on performance, egg quality and fatty acid composition of egg yolk. J. Appl. Anim. Res..

[bib0012] Ceylan N., Ciftci I., Mizrak C., Kahraman Z., Efil H. (2011). Influence of different dietary oil sources on performance and fatty acid profile of egg yolk in laying hens. J. Anim. Feed Sci..

[bib0013] Cherian G., Hayat Z. (2009). Long-term effects of feeding flaxseeds on hepatic lipid characteristics and histopathology of laying hens. Poult. Sci..

[bib0014] Cherian G., Sim J.S. (1991). Effect of feeding full fat flax and canola seeds to laying hens on the fatty acid composition of eggs, embryos, and newly hatched chicks. Poult. Sci..

[bib0015] Cherian G., Traber M.G., Goeger M.P., Leonard S.W. (2007). Conjugated linoleic acid and fish oil in laying hen diets: effects on egg fatty acids, thiobarbituric acid reactive substances, and tocopherols during storage. Poult. Sci..

[bib0016] Coorey R., Novinda A., Williams H., Jayasena V. (2015). Omega-3 fatty acid profile of eggs from laying hens fed diets supplemented with chia, fish oil, and flaxseed. J. Food Sci..

[bib0017] Couëdelo L., Boú -Vaysse C., Fonseca L., Montesinos E., Djoukitch S., Combe N., Cansell M. (2011). Lymphatic absorption of α-linolenic acid in rats fed flaxseed oil-based emulsion. Br. J. Nutr..

[bib0018] Dunbar B.S., Bosire R.V., Deckelbaum R.J. (2014). Omega 3 and omega 6 fatty acids in human and animal health: an African perspective. Mol. Cell. Endocrinol..

[bib0020] Ehr I.J., Persia M.E., Bobeck E.A. (2017). Comparative omega-3 fatty acid enrichment of egg yolks from first-cycle laying hens fed flaxseed oil or ground flaxseed. Poult. Sci..

[bib0021] Faitarone A.B.G., Garcia E.A., Roça R.O., Andrade E.N., Vercese F., Pelícia K. (2016). Yolk color and lipid oxidation of the eggs of commercial white layers fed diets supplemented with vegetable oils. Brazilian J. Poult. Sci..

[bib0022] Feng D., Shen Y., Chavez E.R. (2003). Effectiveness of different processing methods in reducing hydrogen cyanide content of flaxseed. J. Sci. Food Agric..

[bib0023] Folch J., Lees M., Sloane Stanley G.H. (1957). A simple method for the isolation and purification of total lipides from animal tissues. J. Biol. Chem..

[bib0024] Fraeye I., Bruneel C., Lemahieu C., Buyse J., Muylaert K., Foubert I. (2012). Dietary enrichment of eggs with omega-3 fatty acids: a review. Food Res. Int..

[bib0025] García-Rebollar P., Cachaldora P., Alvarez C., De Blas C., Méndez J. (2008). Effect of the combined supplementation of diets with increasing levels of fish and linseed oils on yolk fat composition and sensorial quality of eggs in laying hens. Anim. Feed Sci. Technol..

[bib0026] Goyal A., Sharma V., Upadhyay N., Gill S., Sihag M. (2014). Flax and flaxseed oil: an ancient medicine & modern functional food. J. Food Sci. Technol..

[bib0027] Grčević M., Kralik Z., Kralik G., Galović D., Radišić Ž., Hanžek D. (2019). Quality and oxidative stability of eggs laid by hens fed marigold extract supplemented diet. Poult. Sci..

[bib0028] Horrocks L.A., Yeo Y.K. (1999). Health benefits of docosahexaenoic acid (DHA). Pharmacol. Res..

[bib0029] Imran M., Anjum F.M., Nadeem M., Ahmad N., Khan M.K., Mushtaq Z., Hussain S. (2015). Production of Bio-omega-3 eggs through the supplementation of extruded flaxseed meal in hen diet. Lipids Health Dis.

[bib0030] Jia W., Slominski B.A. (2010). Means to improve the nutritive value of flaxseed for broiler chickens: the effect of particle size, enzyme addition, and feed pelleting. Poult. Sci..

[bib0031] Jia W., Slominski B.A., Guenter W., Humphreys A., Jones O. (2008). The effect of enzyme supplementation on egg production parameters and omega-3 fatty acid deposition in laying hens fed flaxseed and canola seed. Poult. Sci..

[bib0032] Kim M.J., Jung U.S., Jeon S.W., Lee J.S., Kim W.S., Lee S.B., Kim Y.C., Kim B.Y., Wang T., Lee H.G. (2016). Improvement of milk fatty acid composition for production of functional milk by dietary phytoncide oil extracted from discarded pine nut cones (*Pinus koraiensis*) in holstein dairy cows. Asian-Australasian J. Anim. Sci..

[bib0033] Lawlor J.B., Gaudette N., Dickson T., House J.D. (2010). Fatty acid profile and sensory characteristics of table eggs from laying hens fed diets containing microencapsulated fish oil. Anim. Feed Sci. Technol..

[bib0034] Lee K.W., Lillehoj H.S., Jang S.I., Li G.X., Bautista D.A., Phillips K., Ritter D., Lillehoj E.P., Siragusa G.R. (2011). Effects of coccidiosis control programs on antibody levels against selected pathogens and serum nitric oxide levels in broiler chickens. J. Appl. Poult. Res..

[bib0035] Lee S.A., Whenham N., Bedford M.R. (2019). Review on docosahexaenoic acid in poultry and swine nutrition: consequence of enriched animal products on performance and health characteristics. Anim. Nutr..

[bib0036] Lemahieu C., Bruneel C., Termote-Verhalle R., Muylaert K., Buyse J., Foubert I. (2013). Impact of feed supplementation with different omega-3 rich microalgae species on enrichment of eggs of laying hens. Food Chem.

[bib0037] Liang L., Chen F., Wang X., Jin Q., Decker E.A., McClements D.J. (2017). Physical and oxidative stability of flaxseed oil-in-water emulsions fabricated from sunflower lecithins: impact of blending lecithins with different phospholipid profiles. J. Agric. Food Chem..

[bib0038] National Research Council (1994). Nutrient Requeriments of Poultry.

[bib0039] Neijat M., Ojekudo O., House J.D. (2016). Effect of flaxseed oil and microalgae DHA on the production performance, fatty acids and total lipids of egg yolk and plasma in laying hens. Prostaglandins Leukot. Essent. Fat. Acids.

[bib0040] Oliveira D.D., Baião N.C., Cançado S.V., Grimaldi R., Souza M.R., Lara L.J.C., Lana A.M.Q (2010). Effects of lipid sources in the diet of laying hens on the fatty acid profiles of egg yolks. Poult. Sci..

[bib0041] Omri B., Alloui N., Durazzo A., Lucarini M., Aiello A., Romano R., Santini A., Abdouli H. (2019). Egg yolk antioxidants profiles: effect of diet supplementation with linseeds and tomato-red pepper mixture before and after storage. Foods.

[bib0042] Petrović M., Gačić M., Karačić V., Gottstein Ž., Mazija H., Medić H. (2012). Enrichment of eggs in n-3 polyunsaturated fatty acids by feeding hens with different amount of linseed oil in diet. Food Chem.

[bib0043] Pires P.G.S., Leuven A.F.R., Franceschi C.H., Machado G.S., Pires P.D.S., Moraes P.O., Kindlein L., Andretta I. (2020). Effects of rice protein coating enriched with essential oils on internal quality and shelf life of eggs during room temperature storage. Poult. Sci..

[bib0044] Raatz S.K., Redmon J.B., Wimmergren N., Donadio J.V., Bibus D.M. (2009). Enhanced absorption of n-3 fatty acids from emulsified compared with encapsulated fish oil. J. Am. Diet. Assoc..

[bib0045] Rostami A., Zamani Moghaddam A.K., Hassanpour H., Khajali F. (2016). Pulmonary hypertension and right ventricular failure in broiler chickens reared at high altitude is affected by dietary source of n-6 and n-3 fatty acids. J. Anim. Physiol. Anim. Nutr. (Berl)..

[bib0046] Samman S., Kung F.P., Carter L.M., Foster M.J., Ahmad Z.I., Phuyal J.L., Petocz P. (2009). Fatty acid composition of certified organic, conventional and omega-3 eggs. Food Chem.

[bib0047] Shin, B. S., and N. S. An. 2010. Feed Additive Composition Containing Aqueous Flaxseed Oil Without Bad Smell and Cyanide (-CN) Toxicity for Producing Products Containing Omega-3 Fatty Acid: WO 2010/101349 A2.

[bib0048] Thompson M., Hein N., Hanson C., Smith L.M., Anderson-Berry A., Richter C.K., Bisselou K.S., Appiah A.K., Kris-Etherton P., Skulas-Ray A.C., Nordgren T.M. (2019). Omega-3 fatty acid intake by age, gender, and pregnancy status in the United States: national health and nutrition examination survey 2003–2014. Nutrients.

[bib0049] Van Soest P.J., Robertson J.B., Lewis B.A. (1991). Methods for dietary fiber, neutral detergent fiber, and nonstarch polysaccharides in relation to animal nutrition. J. Dairy Sci..

[bib0050] Vlčková J., Tůmová E., Míková K., Englmaierová M., Okrouhlá M., Chodová D. (2019). Changes in the quality of eggs during storage depending on the housing system and the age of hens. Poult. Sci..

[bib0051] Wang Q., Jin G., Wang N., Guo X., Jin Y., Ma M. (2017). Lipolysis and oxidation of lipids during egg storage at different temperatures. Czech J. Food Sci..

[bib0052] Yamashita T., Sano T., Hashimoto T., Kanazawa K. (2007). Development of a method to remove cyanogen glycosides from flaxseed meal. Int. J. Food Sci. Technol..

[bib0053] Yi H., Cho H., Hwang K.T., Shin B.S. (2015). Physical and oxidative stability of flaxseed oil-fructooligosaccharide emulsion. J. Food Process. Preserv..

[bib0054] Yi H., Hwang K.T., Regenstein J.M., Shin S.W. (2014). Fatty acid composition and sensory characteristics of eggs obtained from hens fed flaxseed oil, dried whitebait and/or fructo-oligosaccharide. Asian-Australasian J. Anim. Sci..

